# Specific Prescribing as against Pathological Prescribing

**Published:** 1882-02

**Authors:** P. P. Wells

**Affiliations:** Brooklyn, N. Y.


### SPECIFIC PRESCRIBING AS AGAINST PATHOLOGICAL PRESCRIBING.

P. P. Wells, M. D., Brooklyn, N. Y.

Truth, in all its manifestations, is simple. It is without combination or complexity. It needs no accessories or associates to aid in accomplishing the object for which it was ordained. In itself it is equal to the complete performance of all functions which Supreme Wisdom has imposed upon it. The thought that it needs aught of aid from whatever of outside fact or influence for gaining its true end, can only come from the ignorance or impudence inhering in the folly of beings who are only finite. It is the act of such to reject truth, because of its simplicity, or to attempt to add to it something of their own, imagining thereby to secure a greater power, and therefore a greater success in attaining truth's natural objective. So they commit the great mistake of undervaluing the power of natural simplicity, while at the same time they fall into the greater of overestimating their own ability to add to the efficiency of divine enactments by contributions from their own short-sighted vanity, or presumption. They say this is so simple that it must require something more.

This has been well and abundantly illustrated in the rejection the truth of Nature's law of healing has received from those who insist that more than a discovery of a simillimum of the facts of the case, perceptible to patient and physician, is necessary to the best cure. The law requires similarity of symptoms of disease and drug, nothing more. This is not enough for these people and they will have more. For this, they contend, brings the physician down to a mere "symptom coverer," and this term has been by them cast as a reproach on those who healed by a compliance with Nature's law of healing. Why was this? Because those who were thus derided, did not cure, and cure well? Not so, but because in so complying with law, they left out of the account some notions of these objectors.

Homœopathic prescribing is at all times, in all cases, specific prescribing. That is, finding and giving to the case in hand, the one remedy, in the record of the materia medica, which has been found to have produced on the healthy organism symptoms most like those of the case under treatment. This remedy is found, after examining the case and drawing out all its symptoms, and carefully considering the character and relations of each, by examination and comparison of these with the printed record of the materia medica and in *no other way*. This is the one characteristic peculiarity of homœopathic prescribing as taught by Hahnemann, and which has given to his system of practical medicine a success and reputation which have carried it over the civilized world. It is just this, in its simplicity, which has wrought the great cures which have marked the history of Homœopathy from the beginning. Mixing other notions with this, from whatever source or motive, has been only a damage to practical successes.

"But," says the objector, perhaps a partial homœopathist, "there is in this proceeding no place given to the science of pathology." This is true, but what then? Its absence has not prevented the cure of the sick in the most speedy and sure manner. This is certainly the one great objective of specific prescribing. This greater certainty and celerity is a proof that this valuable science (and it is valuable—it is no part of our intent to underestimate it), is not needed just here, and to insert it where it is not wanted, and more than this, where it does not belong, may prove disastrous. Specific prescribing is the practical use of the science of therapeutics. In this, the science of pathology makes no essential, integral part To thrust it in where it does not belong, in prescribing under our law, is, so far as this has the power, to push back our practice on the old allopathic ground, from the darkness and uncertainty of which we had fondly hoped we had happily escaped. We object to being so put back, and the more, as it is a fact that, just in proportion as this putting back is successful, the ratio of successful curing is diminished. There should be no mistake here. We do not object to the science of pathology, but only to the attempt to impose on it functions which do not belong to it, and which it can in no wise beneficially perform. And in this objection we have reference solely to prescribing under the guidance of our law, and because the law does not include this science among the elements necessary to the right discharge of practical duties under its guidance.

General pathology, like its kin science, pathological anatomy, has its true place among the sciences the enlightened prescriber is expected to know—indeed, with which he is or ought to be quite familiar. And like this kin science, it has its place chiefly in practical diagnosis and prognosis. For example, in painful affections of abdominal organs, by the guidance of the symptoms, and by these alone, the intelligent prescriber decides his case to be inflammatory or neuralgic in its nature, or it may be the result of a strangulated hernia. Thus far pathology may go with the prescriber, and there it stops. It has no word of guidance to the selection of the specific curative for the case. If it be hernia, pathology may suggest the *knife*. A mechanical and dangerous resort. It has no word of any agent which may relieve the sufferer from his pain and danger by a silent force, without bringing into the case any new element of pain or danger. It has no word of suggestion of *Opium* or *Nux vomica*, either of which may and often have superseded the old-time resort of the knife. The same point is illustrated by cases of intestinal intussusception. The pathology of these may be clearly declared by the symptoms of the case, but it has no voice, therefore, to speak of a specific curative for the disease. The most that pathology can ever do for the prescriber, is illustrated by pleurisy. It is a true inflammation, or only a pseudo-imitation of this! This question decided, pathology may suggest two classes of remedies from one of which the specific is to be sought. Thus far it may go and no farther. It has no word of suggestion as to the one remedy.

If this be so, then why the great cry which has recently been revived with so much earnestness, and notably in the late London Convention, of "no pathology"? The reply is found, we believe, in many instances, in the principle with which we opened this paper. The simplicity of the truth, in the matter of prescribing under our law, is so great that some minds are so constituted they will have something more in addition to it. And then this pseudo-pathology (an imaginary internal condition) of the old school was so conveniently at their hand, that their imaginary want was most easily met by seizing on it, and so they rush out and cry aloud their great supposed discovery, and look down on all who are not ready to join their cry, and glorify their pseudo-science by incorporating it into their practical prescribing, though here, indeed, it may well be known it has no place.

Then there is another reason for rejecting our simple truth. It is so grateful to human vanity to find itself looking down on its neighbors, even though it be only from an imaginary height, that the indulgence in the sweetness of it is readily seized on and not easily given up. This addition of an element, so well sounding as this of this pseudo-science, to one's attempts at homœopathic prescribing, puts one at once, in the minds of those who so indulge themselves, on a higher plane than that occupied by their neighbors, who are content with simple truth, and they claim this to be a "scientific addition," and so, why not look down? It is not to be supposed they can help it. Why should they try, indeed, when they have so full a consciousness of having made so important an addition to the work of the master, who had so stamped the thoughts and impress of his genius on the mind of his generation as to effect great and lasting changes in the philosophy and practice of it and of that of all future ages, and so to fix immortality to his name and memory—to add to the works of such a man aught of value, may well feed the vanity of even a good man: The only difficulty here is, the addition is without value to the practical prescriber, and instead of being an improvement of the master's work, is only a blemish.*

If this addition to the master's work, for the sake of the "scientific," were added truth, the case would be different. But it is so often and so largely a creature of the imagination, that it appears there can be no good reason for continued efforts to incorporate this obscuring factor into those needful for finding the simillimum for the sick. It is not helpful in this duty, beyond suggesting a class of remedies from which this is to be selected. The best minds of our school have not made other use of this so-called science, so large a part of which they have clearly seen to be only pseudo-science. So far as it is a necessary part of the factors with which the physician must deal, its usefulness, as we have said, is almost limited to the duties of diagnosis and prognosis. These duties are certainly not unimportant, and in them pathology, true pathology, has a leading part; and for these duties a knowledge of true pathology is indispensable. To pass it beyond this into therapeutics, with which it has so small concern, is, to say the least, a very first-class blunder.

It is worthy of remark that when Hahnemann made his historical prescription for the European typhus which followed the Russian campaign of the first Napoleon, he had no aid from any supposed internal condition, which passed for pathology, but was guided

* The case of the late President, in the hands of those who were regarded as distinguished pathologists, will illustrate the character of very much of what the advocates of this pseudo-science would impose on our homœopathic prescribing, as an addition, it is claimed, from additional knowledge. In the case of the President, it was always guessing, and always guessing wrong. So it is ever.

solely by a knowledge of the symptoms of the disease. The same was true of the Asiatic cholera, when he so truthfully declared its curatives and prophylactics. It has been said he had "no pathology." If this were true, and it is more than likely he had none of the sort now so vaunted, these astonishing successes show he had no need of it, and if he had had it and had made use of it, it is not quite obvious how it could have made his successes greater. In view of these, it is a little singular that those who decry his intelligence, because of his lack of their kind of pathology, do not from these successes learn the little worth there is in it as a contribution to the science of therapeutics. They, certainly, if they would avoid the contempt of all intelligent minds, are not likely to claim greater triumphs in healing, with the aid, whatever it may be, of their imaginary internal condition, than those of the master, without such aid. What is true of Hahnemann in these cases, was and is equally so of that bright constellation of prescribers who were cotemporary with him, and from him drew the light and inspiration which illuminated and governed their practical lives; of this number were Stapf, Gross, Mühlenbein, Bœnninghausen, and others. Do those who claim a superiority of knowledge and practice over these heroes of our school, because of their incorporation of a pseudo pathology into their practice, claim to show, therefore, a better practical record than these? Is it not true of most of those who are loudest in their boasts of their pathology, that these have given us no practical record of their own at all. In the absence of this, and in view of the splendor of that of these heroes of a former time, it may not be untimely to suggest that a becoming modesty on their part will not be regarded as unseemly.

We have given for a title to this paper—"Specific Prescribing as against Pathological Prescribing." One reason for this is that these two methods are in their nature opposed to each other. The first, the specific, deals only with factors which are known and known to be true. It has no concern with the unknown, or with factors which, at best, are only the offspring of conjecture. The facts of the disease, as present to the consciousness of the patient, or the perception of the physician—these are the elements with which it has to do. These are gathered by the prescriber, with all their relations and modalities, and when within his grasp they constitute one side of the equation, which when wrought out gives to him the specific which is the object of his search. It will be seen that these elements are all within the range of the knowable, while the other side is made up of the records of the materia medica, which is a record of facts known. By a comparison of these known quantities, the unknown quantity, *i. e.* the specific, is found. This constitutes specific or homœopathic prescribing. This and nothing else. Its one characteristic is, it deals only with the known.

On the other hand, pathological prescribing deals largely with elements which in whole or in part are objects of conjecture, and therefore are always subject to uncertainty, more or less. The prescriber gathers his facts with more or less completeness, and from these predicates an internal condition, as it may please his fancy to conjecture, and then if he professes any responsibility to the homœopathic law, goes a step further on the same plane of conjecture, to find in the record of the materia medica a medicine which he imagines to have produced in the organism a *condition* similar to that imagined in his patient. This when found and given constitutes the pathological prescribing with which we are at present concerned, and which many affect to dignify with the term "*scientific.*" As science is supposed to be made up of that which is *known*, and this method of prescribing so largely of pure *conjecture*, it is not easy to see just where the science of it comes in, or on what it can found any claim to that expressed by this honorable adjective. If either of the two methods we have described can be characterized by it, it is certainly that which deals with the known, and not that which proceeds so largely on conjecture. For it should be borne in mind, that that *supposed* internal condition which is the peculiar foundation of pathological prescribing, is an unknown and an unknowable quantity during the life of the subject of this conjecture. Omniscience only knows or can know this *exactly*, while the patient lives. The doctor knows it only after the death and dissection of the body of his patient, and then only in part. He only then knows the changes of tissue resulting from the diseased action, and nothing at all of that conjectured condition with which his imagination had been so busy. The action of the invisible laws which make up the diseased state are no more visible in death than in life. Even dissection may fail to show this pseudo-pathologist whether he has rightly guessed or blundered. Omniscience only can ever have *exact* knowledge of the internal condition of the living. The prescriber who pretends to this is either self-deceived or an impostor. Let it be remembered that it is *exact* knowledge which should ever be the objective of the physician's endeavor, and that this is that with which specific prescribing alone concerns itself.

So it will be seen, these two methods of prescribing are the exact opposites of each other. The one dealing exclusively with that which is known, the other so largely with that which is and must be forever unknown to man. It will also be seen how great is the folly of those who would incorporate this pseudo-pathological prescribing into specific prescribing. What can be the benefit of bringing the unknown, or attempting to bring it to the aid of the known. As well attempt to mix ignorance and knowledge, or light and darkness. The two are as alien in spirit as they are different in their methods.*

* The Chinese have a proverb by which they express their sense of the absurdity of an attempt to mix incompatibles, which seems pertinent to efforts to insert this pseudo pathology into homœopathic therapeutics. They say: "You cannot make a partnership between a tailor and a tinker." These have nothing in common: neither have these two antagonistic methods of practice.

